# Fish Oil Supplements Lower Serum Lipids and Glucose in Correlation with a Reduction in Plasma Fibroblast Growth Factor 21 and Prostaglandin E2 in Nonalcoholic Fatty Liver Disease Associated with Hyperlipidemia: A Randomized Clinical Trial

**DOI:** 10.1371/journal.pone.0133496

**Published:** 2015-07-30

**Authors:** Yu Qin, Yong Zhou, Shi-Hui Chen, Xiao-Lan Zhao, Li Ran, Xiang-Long Zeng, Ying Wu, Jun-Li Chen, Chao Kang, Fu-Rong Shu, Qian-Yong Zhang, Man-Tian Mi

**Affiliations:** 1 Chongqing Medical Nutrition Research Center, Chongqing Key Laboratory of Nutrition and Food Safety, Research Center for Nutrition and Food Safety, Institute of Military Preventive Medicine, Third Military Medical University, Chongqing, China; 2 Departments of Health Examination Centre, Southwest Hospital, Third Military Medical University, Chongqing, China; German Diabetes Center, Leibniz Center for Diabetes Research at Heinrich Heine University Duesseldorf, GERMANY

## Abstract

Fish oil has been used effectively in the treatment of cardiovascular disease via triglyceride reduction and inflammation modulation. This study aimed to assess the effects of fish oil on patients with nonalcoholic fatty liver disease (NAFLD) associated with hyperlipidemia. Eighty participants with NAFLD associated with hyperlipidemia were randomly assigned to consume fish oil (n=40, 4 g/d) or corn oil capsules (n=40, 4 g/d) for 3 months in a double-blind, randomized clinical trial. Blood levels of lipids, glucose and insulin, liver enzymes, kidney parameters and cytokines at baseline and the end of the study were measured. Seventy people finished the trial. Plasma concentrations of eicosapentaenoic acid and docosahexaenoic acid significantly increased in the fish oil group after intervention. After adjustment for age, gender and BMI, fish oil significantly decreased fasting serum concentrations of total cholesterol, triglyceride, apolipoprotein B and glucose (by (mean±SD) 0.49±0.43 mmol/L, 0.58±0.89 mmol/L, 0.28±0.33 g/L and 0.76±0.56 mmol/L, respectively, *P*<0.05), as well as alanine aminotransferase and γ-glutamyl transpeptidase levels (by (median (interquartile)) 9.0(0.5, 21.5) and 7.0(2.2, 20.0) IU/L, respectively, *P*<0.05), significantly increased serum adiponectin levels (by 1.29±0.62 μg/mL, *P*<0.001), and reduced serum levels of tumor necrosis factor α, leukotrienes B4, fibroblast growth factor 21 (FGF21), cytokeratin 18 fragment M30 and prostaglandin E2 (by 1.70±1.18 pg/mL, 0.59±0.28 ng/mL, 121±31 pg/mL, 83±60 IU/L and 10.9±2.3 pg/mL, respectively, *P*<0.001). Corn oil had no effect except for increasing serum creatinine concentrations by 7.7±8.9 μmol/L (*P*=0.008). The effects of fish oil on lipids, glucose and γ-glutamyl transpeptidase were positively correlated with the reductions of serum FGF21 and prostaglandin E2 concentrations after adjustment for age, gender and BMI (r = 0.275 to 0.360 and 0.261 to 0.375, respectively, *P*<0.05). In conclusion, our findings suggest that fish oil can benefit metabolic abnormalities associated with NAFLD treatment.

**Trial Registration:**

ChiCTR-TRC-12002380

## Introduction

Nonalcoholic fatty liver disease (NAFLD) comprises a spectrum of liver diseases ranging from simple steatosis, to nonalcoholic steatohepatitis (NASH), to cirrhosis. Recently studies have shown that circulating fibroblast growth factor 21 (FGF21) is positively correlated with the severity and progression of NAFLD [1–3]. The primary source of circulating FGF21 is the liver, and the hepatic synthesis of FGF21 is driven by peroxisome proliferator-activated receptor α activation [4]. High serum FGF21 concentration is an independent predictor of NAFLD in adults [5]. Furthermore, plasma FGF21 levels are significantly and independently correlated with hepatic fat content, markers of hepatic apoptosis and NAFLD scores in obese youths [6]. In addition, previous studies have concluded that plasma levels of cytokeratin 18 (CK18) fragments are correlated with the extent of hepatocyte apoptosis and independently predict the presence of NASH and could thus be used as a noninvasive biomarker for NASH [7].

Fish oil is rich in long-chain omega-3 fatty acids (LC-ω3s) including eicosapentaenoic acid (EPA) and docosahexaenoic acid (DHA). Fish oil supplementation has been used effectively in the treatment of cardiovascular disease (CVD), and the underlying mechanism has been associated with triglyceride reduction and inflammation modulation [8]. NAFLD is associated with an increased risk of atherosclerosis and CVD [9], and fish oil or its constituent long-chain omega-3 fatty acids may be effective in the treatment of NAFLD [10, 11]. Some animal and cell-based studies had demonstrated an ameliorative effect of fish oil and purified LC-ω3s consumption on hepatic lipid content reduction [10]. In addition, dietary supplement of DHA may be effectively in the improvement of liver steatosis in children with NAFLD [12, 13].

However, these previous studies have not focused on the effects of fish oil in adults with NAFLD associated with hyperlipidemia. We conducted a double-blind, randomized clinical trial to assess the effects of fish oil on lipid, glucose and other CVD risk factors, liver enzymes, NAFLD biomarkers such as FGF21 and CK18 fragment M30 (CK18-M30), and some inflammatory cytokines in adults with NAFLD associated with hyperlipidemia.

## Materials and Methods

### Populations

Eighty volunteers with NAFLD characteristics associated with hyperlipidemia were recruited between Sep 2012 and Oct 2013 by distributing leaflets in the streets of Chongqing, China. All participants were enrolled in the trial after a complete physical examination and medical history investigation in the hospital. The inclusion criteria were adults with NAFLD, a steady BMI between 20 and 30 over the last 3 months, and no excessive alcohol consumption (less than 140 g/week for men and 70 g/week for women). A fatty liver was diagnosed by abdominal ultrasonography and identified by characteristic echo patterns including a diffuse increase in the echogenicity of the liver compared with that of the kidney, consistent with conventional criteria [14]. Hyperlipidemia was diagnosed as a fasting serum total cholesterol level higher than 5.2 mmol/L or a fasting serum triglycerides level higher than 1.7 mmol/L [15]. The exclusion criteria were a history of viral hepatitis (including hepatitis B and/or C infection), autoimmune hepatitis or other liver disease; use of any medicine or dietary supplementation in the last 6 months that could influence NAFLD, glucose and lipid metabolism; gastrointestinal disease, severe chronic disease, kidney dysfunctions, or malignant tumors; any acute or chronic infectious disease or injury; or any surgical procedure.

### Study design

This study was a randomized, double-blind, placebo-controlled trial. Eighty eligible people were randomly assigned to the corn oil or the fish oil group and were asked to consume two corn oil or fish oil capsules twice per day for 3 months. The randomized sequence was produced by a randomization protocol using the IBM SPSS Statistics 19.0 (IBM, Japan) system, and the information of randomization was sealed until the end of the study. All participants and people who conducted the trial and assessed outcomes were blinded to the intervention information. All participants were instructed to maintain their habitual dietary style and physical activities during the trial. Each participant visited the hospital at 0 and 3 months, and fasting blood samples were collected for measuring the concentration of parameters such as fatty acid, lipids and liver enzymes. In addition, the height, weight, waist and hip circumferences and blood pressure, triceps skin fold (TSF) of each participant were measured.

The protocol was approved by the Medical Ethics Committee of Third Military Medical University. All participants provided written informed consent. All procedures were in accordance with institutional guidelines and were carried out in compliance with the Helsinki Declaration. This trial was conducted according to "Good Clinical Practice". The registered number of this trial was ChiCTR-TRC-12002380 in the Chinese clinical trial registry (http://www.chictr.org).

The compliance of the participants in the trial was assessed by records of consumed and returned oil capsules and the plasma levels of EPA and DHA. Each subject was asked to record the time and the number of capsules they consumed, and this record was returned to the study team after the trial.

### Interventions

Each fish oil capsule contained 182 mg of EPA and 129 mg of DHA, in addition to vitamin E, gelatin, glycerin and water, and the total weight of each capsule was 1000 mg. The corn oil capsules, the control, contained no EPA or DHA; all other contents were similar to the fish oil capsules. Both oil capsules were prepared and supplied by By-Health Company Limited, Guangdong, China. All capsules were yellow, soft and visually identical and were packaged in sealed gray bottles. All participants consumed two corn oil or fish oil capsules twice per day for 3 months. Thus the total intervention doses per day were 4 g of fish oil capsules, containing a total of 728 mg of EPA and 516 mg of DHA, for participants in the fish oil group, or 4 g corn oil capsules for those in the corn oil group.

### Blood EPA and DHA levels, lipid and glucose metabolism, liver enzymes and kidney parameters and cytokine measurements

Plasma EPA and DHA levels were assayed using gas chromatography methods (Agilent, 6890N, USA) according to the protocol described in the study performed by Masood A and colleagues [16]. The results of plasma EPA and DHA levels were reported as the percentages referred to the total fatty acid. In this study, 12 fatty acids levels in the plasma were tested meanwhile, including C14:0, C16:0, C16:1, C18:0, C18:1, C18:2, C18:3, C20:1, C20:3, C20:4, C20:5(EPA), and C22:6(DHA). The sum of these fatty acids was the total fatty acid in this study.

The primary outcomes were serum fasting total cholesterol and triglyceride concentrations, and the secondary outcomes were serum fasting levels of other lipids (including total, HDL-, and LDL-cholesterol, Apolipoprotein AI and B, and Lipoprotein _(a)_), glucose, and liver enzymes (including alanine aminotransferase (ALT), aspartate transaminase (AST) and γ-glutamyl transpeptidase (GGT)). Assessments of serum fasting lipids, glucose, liver enzymes and kidney parameters (including urea nitrogen, uric acid and creatinine) were performed using routine methods in an automatic biochemical analyzer (Olympus, AU2700, Japan) [17]. Serum insulin and C-peptide concentrations were measured by an electrochemiluminescence immunoassay (Roche) in an automated immuno assay analyzer (Roche, E170, Switzerland). Serum high-sensitivity C-reactive protein (hs-CRP) concentration was assayed by latex enhanced immune turbidimetry using the above analyzer. All the above variables were measured at baseline and at 3 months. The within- and between-assay CVs of all the above methods were < 5%. The homeostasis model assessment of insulin resistance (HOMA-IR) index was calculated as: (fasting glucose concentration × fasting insulin concentration)/22.5.

Serum levels of tumor necrosis factor-alpha (TNF-α), total adiponectin, leukotrienes-B4, CK18-M30, prostaglandin E2 and FGF21 were detected using enzyme-linked immunosorbent assay (ELISA) kits (CUSABIO, USA). All measurements were performed according to the manufacturers’ protocol in duplicate and the within- and between-assay CVs were < 6.7%.

### Statistical analyses

The primary endpoint was the change from baseline (3 month—baseline) in the fasting serum triglyceride. The present trial was designed to provide a greater than 80% statistical power to measure a 0.6 mmol/L reduction in fasting serum triglyceride after 3 months of treatment with fish oil compared with corn oil intervention as the control. The SD of the effects of fish oil or corn oil supplementation was set at 0.75 mmol/L. The significance level was set at 0.05, and 2-tailed tests were used. It was estimated that a sample size of 78 was sufficient to test the primary triglyceride hypothesis while allowing for a 20% dropout rate.

Per protocol statistical method was used. All statistical analyses were performed using SPSS Version 13.0 (SPSS Inc., Chicago, IL). Normally distributed data were expressed as the means ± SDs. Data that were not normally distributed were expressed as median with interquartile range and analyzed after logarithmic transformation. Student’s unpaired t test was used for comparing the levels of anthropometric parameters, serum lipids, glucose, insulin, liver enzymes, liver parameters, cytokines and plasma free fatty acids at baseline, between the two groups. Difference in gender between the 2 groups was assessed by Chi-square tests. The change in each parameter was calculated as the difference between the end value and the baseline value. The effects of fish oil supplements on anthropometric parameters, serum lipids, glucose, insulin, liver enzymes, and cytokines, compared with corn oil supplements, were analyzed using ANCOVA with the change in each parameter as the dependent variable, treatment variable (fish oil or corn oil) as an independent variable, and adjusted with the corresponding values at baseline (Model 1) or adjusted with the corresponding values at baseline, age, gender and BMI (Model 2, as the primary analysis). The reported p-value (for each outcome variable) in ANCOVA was the p-value associated with the treatment variable. Correlation between the effects of fish oil on the serum lipids, glucose, liver enzymes and creatinine and the effects of fish oil on the serum cytokines was assessed by Pearson correlation and partial correlation with adjusted with the corresponding values at baseline, age, gender and BMI. Partial correlation was the primary analysis. Two-sided p values < 0.05 were considered significant.

## Results

### Characteristics of the participants

**Fig 1:**
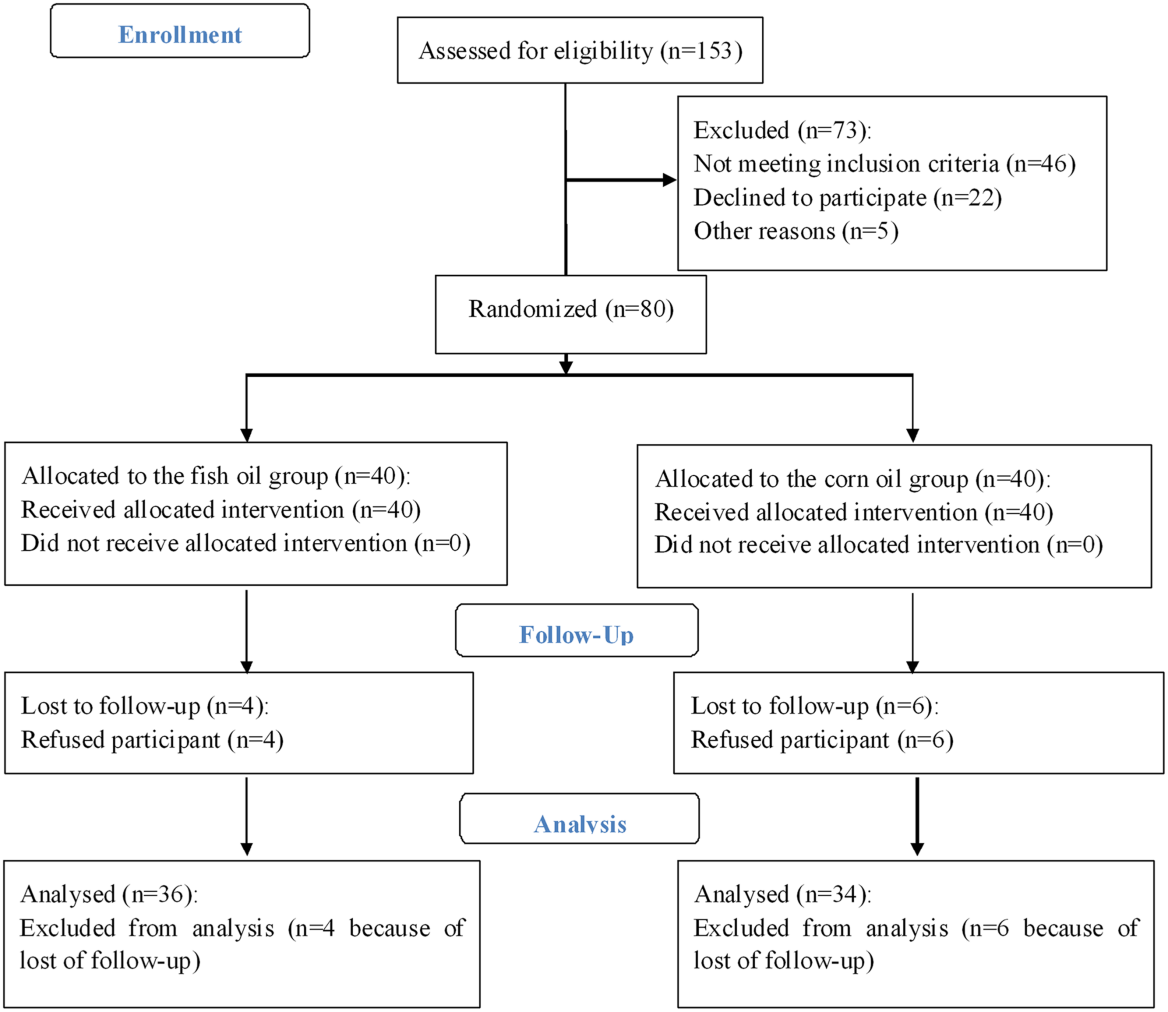
Flow of participants through the trial.

The baseline characteristics of the participants in the fish oil group and the corn oil group were similar (Tables 1 and 2). After the 3 month intervention, seventy subjects (34 and 36 subjects in the corn oil group and fish oil group, respectively) completed this trial (Fig 1).

**Table 1:** Effects of fish oil on anthropometric parameters, serum lipids and glucose metabolism, and EPA and DHA in study participants^a^.

	Corn oil (n = 34)			Fish oil (n = 36)			*P*	ANCOVA^b^	
	Baseline	End	Change^c^	Baseline	End	Change	Baseline	Model 1	Model 2
**Age (y)**	44.3±10.90			46.0±10.68			0.50		
**Male/Female**	25/9			26/10			0.90		
**BMI (kg/m** ^**2**^ **)**	26.0±2.8	26.0±2.8	0.0±0.9	26.4±3.9	26.1±3.8	-0.3±0.7	0.57	0.13	
**Weight (Kg)**	70.4±10.1	70.5±10.2	0.1±2.4	72.9±13.5	72.2±13.8	-0.7±2.0	0.38	0.13	0.18
**Waist C (cm)**	88.9±9.7	87.9±7.7	-1.0±12.6	91.4±10.2	89.0±8.0	-2.4±13.5	0.31	0.54	0.83
**Hip C (cm)**	97.8±5.8	98.7±6.6	0.9±5.6	100.1±7.4	100.0±7.2	-0.1±3.6	0.14	0.56	0.72
**WHR**	0.91±0.13	0.89±0.06	-0.02±0.14	0.91±0.07	0.89±0.10	-0.02±0.15	0.91	0.86	0.86
**TSF (mm)**	28.1±2.5	27.6±2.8	-0.4±2.5	27.7±2.9	27.1±2.6	-0.6±2.4	0.58	0.48	0.58
**SBP (mmHg)**	130±21	131±23	1±15	128±17	132±20	4±13	0.59	0.36	0.42
**DBP (mmHg)**	84±15	84±15	0±11	82±12	83±11	1±7	0.54	0.73	0.85
**TC (mmol/L)**	5.40±1.08	5.46±1.05	0.06±0.75	5.33±1.18	4.84±1.05	-0.49±0.43	0.79	<0.001	<0.001
**TG (mmol/L)**	1.90±1.04	1.95±1.21	0.04±0.88	2.12±1.03	1.53±0.85	-0.58±0.89	0.38	0.006	0.005
**LDL-C(mmol/L)**	3.29±0.74	3.10±0.68	-0.19±0.74	3.27±0.66	2.94±0.67	-0.32±0.47	0.88	0.27	0.26
**HDL-C (mmol/L)**	1.27±0.24	1.30±0.27	0.03±0.24	1.27±0.19	1.32±0.22	0.05±0.17	0.96	0.63	0.69
**ApoA-I (g/L)**	1.51±0.18	1.52±0.25	0.01±0.33	1.57±0.23	1.60±0.26	0.03±0.34	0.20	0.17	0.17
**ApoB (g/L)**	1.24±0.31	1.24±0.26	0.00±0.45	1.34±0.30	1.06±0.23	-0.28±0.33	0.14	0.006	0.007
**Lipoprotein (a) (mg/L)**	162 (135, 219)	159 (116, 219)	-21(-66, 52)	168(128, 254)	169(101, 251)	-20(-43, 18)	0.45	0.67	0.62
**Glucose (mmol/L)**	5.68±1.10	5.46±0.68	-0.21±1.41	5.93±0.56	5.17±0.44	-0.76±0.56	0.24	0.041	0.048
**Insulin (mIU/L)**	11.64(8.97, 15.85)	11.31(8.68, 14.43)	-1.47(-3.05, 1.66)	11.95(10.46, 16.54)	13.24(10.52, 16.08)	0.53(-1.58, 2.11)	0.81	0.18	0.16
**HOMA-IR** ^**d**^	2.84(2.30, 3.95)	2.80(2.07, 3.51)	-0.25(-0.75, 0.25)	3.15(2.63, 4.09)	2.99(2.37, 3.75)	-0.25(-0.85, 0.30)	0.36	0.78	0.67
**C-peptide (ng/ml)**	4.40±1.12	4.04±1.16	-0.36±1.00	4.34±0.97	3.89±1.13	-0.44±0.79	0.80	0.62	0.58
**EPA (%)**	0.68±0.04	0.69±0.04	0.01±0.03	0.68±0.04	3.48±0.29	2.80±0.28	0.67	<0.001	<0.001
**DHA (%)**	2.33±0.20	2.38±0.35	0.05±0.36	2.32±0.20	4.33±0.28	2.01±0.28	0.51	<0.001	<0.001

^a^ Data are n or mean±SD or median (interquartile range).

Abbreviations: ApoA-I: apolipoprotein A-I; ApoB: apolipoprotein B; BMI: body mass index; DBP: diastolic blood pressure; DHA: docosahexaenoic acid; EPA: eicosapentaenoic acid; HDL-C: high-density lipoprotein cholesterol; Hip C: hip circumference; HOMA-IR: homeostasis model assessment insulin resistance index; LDL-C: low-density lipoprotein cholesterol; SBP: systolic blood pressure; TSF: triceps skin fold; TC: total cholesterol; TG: triglyceride; Waist C: waist circumference; WHR: ratio of waist to hip circumference.

^b^ Model 1: adjusted by the baseline levels. Model 2: adjusted by the baseline levels, age, gender and BMI.

^c^ Change is equal to the end values—the baseline values.

^d^ HOMA-IR equaled to (fasting glucose concentration × fasting insulin concentration)/22.5.

**Table 2:** Effects of fish oil on blood liver enzymes, kidney parameters and cytokines in study participants^a^.

	Corn oil (n = 34)			Fish oil (n = 36)			*P*	ANCOVA^b^	
	Baseline	End	Change^c^	Baseline	End	Change	Baseline	Model 1	Model 2
**ALT (IU/L)**	33.0(27.0, 47.0)	28.0(23.7, 45.0)	-5.0(-21.0, 7.5)	31.5(25.0, 51.2)	25.5(21.2, 30.0)	-9.0(-21.5, -0.5)	0.78	0.018	0.027
**AST (IU/L)**	27.5(24.0, 33.2)	28.0(22.0, 32.0)	-1.0(-6.0, 3.0)	28.0(23.2, 34.7)	27.0(21.0, 30.7)	-2.0(-6.0, 2.0)	0.85	0.62	0.64
**GGT (IU/L)**	40.0(24.0, 73.0)	35.0(27.7, 47.2)	-3.5(-11.2, 3.0)	34.0(24.2, 66.0)	24.5(19.0, 42.2)	-7.0(-20.2, -2.0)	0.96	0.001	0.001
**Urea nitrogen (mmol/L)**	5.02±1.14	5.36±1.04	0.34±1.34	5.15±0.96	5.35±1.30	0.20±1.05	0.62	0.78	0.61
**Uric acid (μmol/L)**	392±80	381±81	-11±63	403±104	370±93	-33±55	0.61	0.14	0.07
**Creatinine (μmol/L)**	73.3±16.0	81.0±14.9	7.7±8.9	76.6±14.0	78.0±13.1	1.4±8.8	0.36	0.006	0.008
**hs-CRP (mg/L)**	1.63(0.95, 2.96)	1.41(0.56, 2.97)	-0.24(-0.60, 0.48)	1.63(0.92, 3.52)	1.83(0.73, 3.25)	-0.17(-0.62, 0.55)	0.54	0.57	0.69
**Adiponectin (μg/mL)**	5.05±0.58	5.17±0.80	0.12±0.99	5.08±0.46	6.37±0.40	1.29±0.62	0.80	<0.001	<0.001
**TNF-α (pg/mL)**	4.78±0.87	4.59±0.91	-0.21±1.11	4.52±0.71	2.82±0.96	-1.70±1.18	0.19	<0.001	<0.001
**LT-B4 (ng/mL)**	0.94±0.28	0.70±0.25	-0.23±0.36	0.94±0.25	0.35±0.10	-0.59±0.28	0.97	<0.001	<0.001
**FGF21 (pg/mL)**	236±29	206±44	-30±58	237±28	115±10	-121±31	0.90	<0.001	<0.001
**CK18-M30 (IU/L)**	271±45	270±42	-1±61	283±55	200±26	-83±60	0.32	<0.001	<0.001
**PG-E2 (pg/mL)**	42.4±1.2	38.0±3.9	-4.4±4.1	42.1±1.2	31.2±2.0	-10.9±2.3	0.46	<0.001	<0.001

^a^ Data are mean±SD or median (interquartile range).

Abbreviations: ALT: alanine aminotransferase; AST: aspartate transaminase; CK18-M30: cytokeratin 18 fragments M30; FGF 21: fibroblast growth factor 21; GGT, γ-glutamyl transpeptidase; hs-CRP: high-sensitivity C-reactive protein; LT-B4: Leukotrienes B4; PG-E2: Prostaglandin E2; TNF-α: Tumor necrosis factor-alpha.

^b^ Model 1: adjusted by the baseline levels. Model 2: adjusted by the baseline levels, age, gender and BMI.

^c^ Change is equal to the end values—the baseline values.

### Compliance

Ten subjects withdrawn from the study, including 6 persons refused to go back to hospital at the end of the trial, and 4 persons did not answer our contact. No adverse reactions were reported by the participants during the trial.

The compliance of all subjects was good. The rate of capsules consumed was 98.9% in the corn oil group, and 99.2% in the fish oil group. In the fish oil group, the plasma EPA and DHA levels significantly increased after the trial (both P < 0.05 compared with baseline and the corn oil group). In the corn oil group, the levels of EPA and DHA did not change significantly. These data had been presented as Table 1 (EPA and DHA). Plasma levels of other free fatty acids were presented in S1 Table. In the corn oil group, the plasma linoleic acid (C18:2, a major content in the corn oil) levels increased after the intervention.

### Anthropometric parameters, serum lipids and glucose metabolism of the participants

Compared to the corn oil intervention, fasting serum total cholesterol, triglyceride, apolipoprotein B and glucose concentrations of the participants decreased significantly after fish oil intervention for 3 months (Table 1). There was no significant difference between the effects of fish oil and corn oil supplementation on anthropometric parameters, fasting serum LDL-C, HDL-C, apolipoprotein A-I, lipoprotein (a), insulin and C-peptide concentrations, and HOMA-IR index. Adjustments for age, gender and BMI at baseline did not alter this result.

### Liver enzymes, kidney parameters of the participants

Compared to the corn oil intervention, serum ALT and GGT levels were significantly reduced after fish oil supplementation for 3 months (Table 2). The increase in serum creatinine concentration was smaller in the fish oil group than in the corn oil group, i.e. fish oil prevented the increase of creatinine. Serum AST, urea nitrogen, and uric acid concentrations in both groups did not significantly change after the 3 month trial.

### Serum cytokines of the participants

Compared to the corn oil group, fish oil intervention for 3 months significantly increased serum adiponectin levels, and reduced serum TNF-α, leukotrienes-B4, FGF21, CK18-M30, and prostaglandin E2 levels in patients with NAFLD characteristics. Serum hs-CRP levels in the two groups did not significantly change during the trial.

### Correlation between the effects of fish oil on the serum lipids, glucose, liver enzymes and creatinine and the effects of fish oil on the serum cytokines

After adjustment for age, gender and BMI, the reductions in serum total cholesterol, triglyceride, apolipoprotein B, glucose, and log-transformed GGT concentrations were significantly and positively correlated with the reductions of serum FGF21 and prostaglandin E2 levels for all participants (r = 0.275 to 0.360 and 0.261 to 0.375, respectively, P < 0.05, Table 3). Meanwhile, although serum creatinine concentrations increased more in the corn oil group than that in the fish oil group, the change in serum creatinine was also significantly and positively correlated with the changes in serum FGF21 and prostaglandin E2 levels for all participants (r = 0.432 and 0.301, respectively, P < 0.05, Table 3).

**Table 3:** Correlation of the changes of the lipids, glucose, liver enzymes and creatinine concentrations and the changes of the serum cytokines levels of the populations.

		Pearson correlation						Partial correlation					
variables		Adiponectin	TNF-á	LT-B4	FGF21	CK18-M30	PG-E2	Adiponectin	TNF-á	LT-B4	FGF21	CK18-M30	PG-E2
**TC**	r	-0.246	0.165	0.207	0.372	0.135	0.353	-0.242	0.165	0.241	0.360	0.136	0.341
	*P*	0.040	0.172	0.085	0.002	0.265	0.003	0.049	0.181	0.049	0.003	0.271	0.005
**TG**	r	-0.217	0.331	0.260	0.308	0.100	0.240	-0.222	0.323	0.248	0.329	0.088	0.261
	*P*	0.071	0.005	0.030	0.009	0.409	0.046	0.071	0.008	0.043	0.007	0.480	0.033
**Apo B**	r	-0.173	0.266	0.175	0.368	0.257	0.392	-0.166	0.264	0.222	0.356	0.256	0.375
	*P*	0.153	0.026	0.147	0.002	0.032	0.001	0.180	0.031	0.070	0.003	0.036	0.002
**Glucose**	r	-0.219	0.037	0.170	0.298	0.157	0.306	-0.215	0.050	0.217	0.275	0.178	0.291
	*P*	0.069	0.760	0.160	0.012	0.194	0.010	0.081	0.687	0.077	0.024	0.149	0.017
**ALT**_**(log)**_	r	-0.071	0.111	0.284	0.174	0.299	0.139	-0.075	0.104	0.278	0.188	0.303	0.170
	*P*	0.561	0.361	0.017	0.149	0.012	0.252	0.545	0.401	0.023	0.128	0.013	0.168
**GGT**_**(log)**_	r	-0.115	0.230	0.280	0.304	0.297	0.240	-0.131	0.237	0.244	0.350	0.309	0.302
	*P*	0.344	0.056	0.019	0.011	0.012	0.046	0.289	0.054	0.047	0.004	0.011	0.013
**Creatinine**	r	-0.164	0.080	0.304	0.434	0.172	0.294	-0.165	0.093	0.324	0.432	0.190	0.301
	*P*	0.174	0.509	0.011	<0.001	0.156	0.013	0.183	0.454	0.008	<0.001	0.124	0.013

Partial correlations were adjusted with age, gender and BMI. Abbreviations: ApoB: apolipoprotein B; ALT: alanine aminotransferase; CK18-M30: cytokeratin 18 fragments M30; FGF 21: fibroblast growth factor 21; GGT, ã-glutamyl transpeptidase; LT-B4: Leukotrienes B4; PG-E2: Prostaglandin E2; TNF-á: Tumor necrosis factor-alpha.

In addition, the change in serum FGF21 levels was negatively associated with the change in serum adiponectin levels (r = -0.428 without adjustment, and -0.426 with adjustment, P < 0.001).

## Discussion

This study found that 4 g of fish oil consumption for 3 months effectively decreased serum total cholesterol, triglyceride, apolipoprotein B and glucose concentrations in an Asian population with NAFLD associated with hyperlipidemia. In addition, fish oil intervention was effective in the reductions of serum ALT and GGT levels of these participants. Furthermore, fish oil decreased the levels of serum NAFLD biomarkers, CK18-M30 and FGF21. All above results suggest that fish oil supplement can ameliorate liver function and play a role in the treatment of NAFLD. Our results on liver enzymes were similar to those of a meta-analysis conducted by Parker HM and colleagues [18]. They found that omega-3 PUFA supplement decreased liver fat and liver function including AST and ALT.

Our study also demonstrated that the decreases of serum total cholesterol, triglyceride, apolipoprotein B and glucose concentrations and GGT levels induced by fish oil were positively correlated with the reduction of serum FGF21 levels. Several human studies found that circulation FGF21 levels was positively associated with the degree of hepatic steatosis, type 2 diabetes mellitus (T2DM), and obese [19–22]. The anti-diabetes treatment often produced a reduction of circulating FGF21 levels in T2DM patients [20, 21]. However, animals studies reported that increasing FGF21 or FGF21 treatment had a beneficial effect on glucose and lipid metabolism, weight loss and NAFLD [23–25]. The paradox effects of FGF21 on metabolic regulation between humans and animals may be due to FGF21 resistance existed in humans with NAFLD, obese and T2DM [3, 26]. Treatment of hepatic or systemic FGF21 resistance maybe important in the treatment of NAFLD and other diseases, and this FGF21 resistance state can be ameliorated by certain therapies. For example, Samson SL and colleagues found that combined treatment with pioglitazone and exenatide decreased circulation FGF21 levels, hepatic FGF21 protein and mRNA, and hepatic fat in a mouse model of obesity and in obese patients with T2DM [27]. In our study, fish oil treatment markedly reduced circulating biomarker of NAFLD, FGF21 levels, combined with reductions of serum lipids, glucose, liver enzymes, and other NAFLD risk factor, cytokeratin 18 fragment M30. These results suggested that fish oil may have an effect on the improvement of FGF21 resistance. However, to date, few studies in humans reported the effects of fish oil or LC-ω3s on the circulating NAFLD biomarkers, FGF21 and CK18 fragments. In mice, dietary LC-ω3s prevented the increase of plasma FGF21 levels induced by high-fat diet [28]. Further studies are needed to confirm the beneficial effect of fish oil on the FGF21 resistance in NAFLD, and to investigate the underlying mechanisms for FGF21 as a therapy target of NAFLD and other diseases treatment.

In addition, fish oil markedly reduced circulating levels of inflammatory cytokines such as TNF-α and leukotrienes B4, and the pro-inflammatory cytokine prostaglandin E2, and increased adiponectin. A number of cellular and animal studies have demonstrated that LC-ω3s exert their anti-inflammatory effects by a reduction in the activation of nuclear factor-κB [29]. Previous studies found that fish oil supplement significantly increased the circulating adiponectin levels in humans [30–32]. Circulating adiponectin levels in obese humans were lower than that in lean humans, and increased after weight loss by the surgery or low-calorie diet in obese humans [33, 34]. However, fish oil supplement lead a significant increase of circulating adiponectin levels, companied with a small reduction of weight (mean 0.75 Kg) without significant in our study. Our results were similar to others. Gammelmark et al found that low-dose fish oil supplementation increased serum adiponectin with no change in weight in overweight subjects [35]. In addition, a meta-analysis included 15 randomised clinical trials found that participants taking fish or fish oil significantly lost 0.59 kg more body weight than controls (95% confidence interval: −0.96 to −0.21) [36], which degree of weight loss was similar to that in our study. Our finding suggested that the increase of circulating adiponectin levels in subjects consumed fish oil supplementary may not only due to the reduction of weight. The mechanism underlying the effects of fish oil on adiponectin should be investigated in the further studies.

In this study, the change in serum FGF21 levels induced by fish oil supplement was negatively associated with the change in serum adiponectin levels. Our finding was different from that in animal studies, but similar to other human studies. Previous animal studies found that FGF21 once intravenous infused or daily FGF21 intraperitoneal injection for 12 weeks induced an increase of adiponectin in mice [23, 37, 38]. However, circulating FGF21 levels were negative or no association with adiponectin levels in humans. Mraz et al found that serum adiponetin levels were negatively associated with serum FGF21 levels in both obese and T2DM patients [39]. However, other studies did not reveal the correlation between FGF21 and adiponectin in adults with T2DM [40] and in healthy Danish children [41]. In our opinion, different association of the 2 parameters between animals and humans may be due to the following factors: (a) different between animal experiments and human studies. (b) FGF21 directly treatment through intraperitoneal injection or infusion often produced higher circulating levels of FGF21 (about 100 to 8000 times) than that increased by some medicine or chemicals in humans. (c) Other factors such as different diseases. Therefore, a relationship between FGF21 and adiponectin was still unclear.

Our study also demonstrated that the beneficial effects of fish oil on the serum lipids, glucose and GGT levels were positively related to serum prostaglandin E2 concentrations reduction. Previous studies have shown that fish oil or LC-ω3 intervention effectively reduces triglyceride levels and improves anti-inflammation functions [8]. These effects of LC-ω3s are mediated in part by antagonizing ω-6 polyunsaturated fatty acid (arachidonic acid)-induced pro-inflammatory prostaglandin E2 formation [29]. Thus, the mechanism by which fish oil improves lipid levels and anti-inflammation functions may partly through the inhibition of the production of prostaglandin E2.

Furthermore, serum creatinine concentrations significantly increased after the corn oil supplementation for 3 months, while not significantly changed after the fish oil intervention. This “negative” effect of the corn oil on serum creatinine may be associated with the higher levels of plasma linoleic acid. Our finding was different from the finding of Wong CY and colleagues [42]. They found that 3 months of daily 4 g of fish oil supplement decreased serum creatinine concentrations by 3.3 μmol/L compared with olive oil supplement in patients with T2DM [42]. The paradoxical conclusion may attribute to the different participants. Our subjects were adults with NAFLD accompanied with hyperlipidemia with a mean age of 45 years. The patients participated in Wong’s study were adults with T2DM with a mean age of 60 years, those may have potential kidney dysfunctions even without observed clinical manifestations. Fish oil supplement may easily perform the beneficial effects on the kidney functions in people with potential dysfunctions in kidney. Overall, 4 g of fish oil supplementation for 3 months did not harm the kidney function in the above people.

Our study has several limitations. First, the patients with NAFLD used in this study were only diagnosed by B ultrasonic testing and not by liver biopsy or magnetic resonance image (MRI). The reasons for the use of abdominal ultrasonography examination to diagnose NAFLD were that it was noninvasive and our volunteers did not accept liver biopsy. In addition, the cost of MRI was very expensive. Secondly, we did not assess the effect of fish oil on the liver fat content. Previous studies had concluded that fish oil had the ability to decrease the liver fat content. A meta-analysis included 9 clinical studies and conducted by Parker et al found that LC-ω3s consumption effectively reduced the liver fat [18]. Thirdly, we only assessed the hepatitis B and/or C infection of participants by enquiring infection history, instead of testing antibody or antigen levels. Any people with a history of the hepatitis B and/or C infection were excluded from the trial according to the protocol. However, the method of the hepatitis B and/or C infection test may not change our conclusion significantly. Finally, the short duration could not demonstrate the long-term effects of fish oil consumption in adults with NAFLD. Further studies are needed to confirm the findings in the current study.

## Conclusion

Fish oil supplements (4 g) for 3 months improved lipid and glucose levels, liver function and the circulating biomarkers FGF21 and CK18 fragment, and performed anti-inflammation functions in patients with NAFLD associated with hyperlipidemia. The effects of fish oil on lipid and glucose levels and liver functions were positively correlated with the reductions of FGF21 and prostaglandin E2. Our findings suggest that supplementation with fish oil can have benefits in the treatment of metabolic abnormalities associated with NAFLD.

## Supporting Information

S1 CONSORT Checklist CONSORT 2010 checklist. (DOCX)

S1 Protocol The protocol of fish oil trial. (DOCX)

S1 Table Effects of fish oil or corn oil on plasma free fatty acids spectra in study participants. (DOCX)
