# Supplementary material for: Fish Oil Supplements Lower Serum Lipids and Glucose in Correlation with a Reduction in Plasma Fibroblast Growth Factor 21 and Prostaglandin E2 in Nonalcoholic Fatty Liver Disease Associated with Hyperlipidemia: A Randomized Clinical Trial
Source: PLoS One. 2015 Jul 30;10(7):e0133496. doi: 10.1371/journal.pone.0133496 (PMC4520650; doi:10.1371/journal.pone.0133496)
Supplement: S1 Protocol — (DOCX) [file pone.0133496.s002.docx]

The protocol of fish oil trial

The protocol of the fish oil trail was originally published in Chinese, and was also translated to English here.

中文版本（The Chinese version）：

题目：鱼油对非酒精性脂肪性肝病患者营养干预效应研究

1 研究对象的选择

在重庆市沙坪坝区常住居民中募集患有非酒精性脂肪性肝病（NAFLD）的志愿者。受试人员经课题组人员的解释和说明后，自愿参加本研究并签署知情同意书。

纳入标准：符合以下所有条件者，可以纳入试验。①年龄在20-60岁，重庆市常住居民（在重庆市居住5年以上者）；②B超和肝功检查诊断为中度NASH且未使用药物治疗者；③BMI>20且<30，近三个月内体重稳定；④每周饮用酒精量在男性<140g，女性<70g。

排除标准：有下列情况之一者，则排除试验。①肝炎病毒感染者。②近半年内服用过任何明确影响血脂、血糖代谢的药物。③患有胃肠道疾病、各种严重的慢性疾病或肝肾功能不全、恶性肿瘤的患者。④研究前一个月内曾患有急慢性感染性疾病、外伤或手术。⑤依从性差的患者（即不能保证完成12个月的干预和复诊）。

2 试验设计

采用随机、双盲、平行对照试验设计。

（1）样本量估计

本研究为平行对照试验设计，按照相应公式计算研究所需样本量。考虑20%的失访率，每组需要40名研究对象，共需80名研究对象。

（2） 试验分组及试验实施

① 试验分组和盲法实施：符合要求的研究对象，SPSS软件产生随机序列，将研究对象随机分入对照组和鱼油组。随机序列保持在密封信封中，在试验结束后揭盲。受试人员和研究人员均不知道试验分组情况。

② 干预方法与干预周期：所有研究对象均接受营养教育和营养指导。各干预品均制成统一胶囊包装，外形、内容物都无明显外观差异，重量一致，只是对照组不含鱼油，鱼油组的胶囊每粒含1g鱼油。早晚各服1次，每次2粒。向参与试验人员一次分发2周样品，并嘱咐其按要求登记服用情况。每次发放时询问和检查前一次服用干预品的情况，了解患者有无不良反应及其种类。干预3个月。

③ 随访：干预3个月时，完成相关医学检查、膳食调查，收集研究对象清晨空腹血样用于指标检测。

3 基本情况调查

采用统一的调查表，经统一培训的调查员面对面询问研究对象，并根据其回答如实填写问卷。所有研究对象进行基本情况、健康状况、生活方式调查，主要内容包括：① 基本情况：姓名、年龄、性别等；② 基本人口社会学特征：职业、教育程度、婚姻状况、家庭收入；③ 生活方式：饮酒、吸烟、运动情况、饮茶等；④ 疾病史和家族史；⑤服用药物和营养补充剂(包括多酚类保健品或补充剂、抗氧化类维生素，如大豆异黄酮、茶多酚、维生素E等)情况等。

4 膳食调查与膳食控制

膳食调查：选用本实验室制定的针对重庆地区居民的半定量食物频数问卷(SQFFQ)作为膳食评价工具，同时结合膳食记录法和称量法进行膳食调查。所有研究对象在干预前接受食物频数问卷调查，由专职调查员统一询问受试对象近1年来的膳食摄入情况。

干预试验期间的膳食控制：原则上在整个干预期间研究对象应尽量遵守我们提供的NAFLD膳食指导原则。另外忌服各种形式含植物多酚类的营养补品或保健食品。

5 主要观察指标

（1）鱼油干预对NAFLD患者体脂含量及分布影响

一般医学指标检测：测量身高、体重、腰围、臀围、上臂围、皮褶厚度和血压，并计算体质指数（BMI）和腰臀比（WHR）。

（2）鱼油干预对NAFLD患者肝功的影响

在第三军医大学西南医院、新桥医院健康体检中心采用全自动生化仪检测。重点分析ALT、AST等指标。

（3）鱼油干预对NAFLD患者糖脂代谢的影响

① 脂代谢指标：包括空腹血清TC、LDL-C、HDL-C、TG、apoAI、apoB、游离脂肪酸等。TC、TG、LDL-C、HDL-C、apoAⅠ、apoB均用全自动生化仪检测；游离脂肪酸水平采用比色法测定。

② 糖代谢指标：包括空腹血糖、胰岛素水平和HOMA-IR等。血糖采用葡萄糖氧化酶法测定，胰岛素水平采用ELISA法，计算HOMA-IR=空腹血糖（mmol/L）×胰岛素（μIU/ml）/22.5。

1. 试验完成后的统计结果：

所有统计分析使用SPSS 软件。符合正态分布的数据表示为均数±标准差。不符合正态分布的数据表示为中位数和四分位范围，对数转换后进行分析。在基线和结束时两组之间的人体测量参数的水平、血脂、葡萄糖、胰岛素、肝酶等指标的差异采用t检验。两组间构成比之间的差异采用卡方检验。参数的变化值等于结束时的值减去基线值。鱼油补充对人体测量参数、血脂、葡萄糖、胰岛素,肝酶等指标的效应采用协方差分析，以干预后的值作为因变量，并以基线值进行校正(模式1)，或基线值、年龄、性别和体重指数进行校正(模型2)。以双侧p值< 0.05认为具有统计学意义。

The English version (英文版本):

Title：The effects of fish oil on patient with non-alcoholic fatty liver disease

1. The selection of populations

Volunteers with non-alcoholic fatty liver disease (NAFLD) characteristics were recruited by distributing leaflet in streets in Chongqing, China. All participants were included into the trial after completely physical examination and medical history investigation in hospitals. All populations provided written informed consent.

The inclusion criteria were adults with NAFLD while no use any medical for the treatment of NAFLD, with age between 20 and 60 years, steady BMI between 20 and 30 last 3 months, and without excessive alcohol consumption (more than 140 g/week for men and 70 g/week for women). A fatty liver was diagnosed by abdominal ultrasonography and identified by characteristic echo patterns including a diffuse increase in the echogenicity of the liver compared with that of the kidney consistent with conventional criteria.

The exclusion criteria were a history of viral hepatitis, autoimmune hepatitis or other liver disease; taken any medicine or dietary supplementation that influence NAFLD, glucose and lipid metabolism last 6 months; suffered from gastrointestinal disease, severe chronic disease, kidney dysfunctions, or malignant tumors; suffered from any acute or chronic infectious diseases or injury, or receive any surgery; patients with poor compliance.

2 Study design

This study was a randomized, double-blind, placebo-controlled trial.

(1) the estimation of the sample size

The present trial was designed to provide a greater than 80% statistical power to measure a 0.6 mmol/L of difference in reduction fasting serum triglycerides after 3 months treatment of fish oil, compared to corn oil intervention as the control. It was estimated that a sample size of 80 was sufficient to test the primary triglycerides hypothesis while allowing for a 20% dropout rate.

(2) the population randomization and trial implementation

Randomization and blinding: eligible people were randomly assigned to the control or the fish oil group, and were asked to consume two corn oil or fish oil capsules twice per day for 3 months. The random sequence was generated by using the SPSS software. The information of randomization was sealed until the end of the study. All participants and people who conducted the trial and assessed outcomes were blinded to the intervention information.

Intervention: All subjects were given the nutritional education and guidance. Each fish oil capsule contained 182 mg of EPA and 129 mg of DHA, the whole weight was 1000 mg in addition with vitamin E, gelatin, glycerin and water. The corn oil capsules as the control contained no EPA or DHA and other contents similar to the fish oil capsules. Both oil capsules were prepared and supplied by By-Health Company Limited, Guangdong, China. All capsules were yellow soft capsules and visually identical and packaged in a sealed gray bottles. All participants consumed two corn oil or fish oil capsules twice per day for 3 months. Thus the total intervention doses per day were 4 g fish oil capsules contained 728 mg of EPA and 516 mg of DHA for participant in the fish oil group, or 4 g corn oil capsules for those in the corn oil group.

Follow-up: All participants were instructed to maintain their habitual dietary style and physical activities. Each participant attended hospital at 0 and 3 months and fasting blood samples were collected for parameters such as fatty acid, lipids and liver enzymes concentrations detecting. In addition, the height, weight, waist circumference and blood pressures of peoples were measured meanwhile.

1. The information collection at baseline

The information of all populations at baseline was collected using formed questionnaire by face to face investigation by the trained investigators. The information collected including: basically information such as name, age and gender; demographic sociology characteristics; life styles such as drinking, smoking and exercise; diseases history and family history; drugs and nutritional supplements use.

4 Dietary investigation and dietary control

The semi-quantitative food frequency questionnaire and dietary food records were used for estimating the dietary energy and nutrients intakes of all population. During the whole intervention, each subject was asked to adhere to our protocol and do not administrate any forms of plant polyphenols nutritional supplements and health food.

5 Measurement of clinical parameters

(1) general medical index detection: measure the height, weight, waist circumference, hip circumference, upper arm circumference and skinfold and blood pressure, and calculate the body mass index (BMI) and waist-to-hip ratio (WHR).

(2) hepatic, kidney function test: including ALT and AST.

(3) the influence of the fish oil intervention on the glucolipid metabolism in patients with NAFLD, including fasting serum TC, TG, LDL-C, HDL-C, apoAI, apoB, free fatty acids, glucose, insulin levels and HOMA IR.

6 Statistical analyses

All statistical analyses were performed using SPSS Version 13.0 (SPSS Inc., Chicago, IL). Normally distributed data were expressed as means ± SDs. Data that
were not normally distributed were expressed as median with interquartile range, and analyzed after logarithmically transformed. Student’s unpaired t test was used for comparison the levels of anthropometric parameters, serum lipids, glucose, insulin, liver enzymes, liver parameters, cytokines at baseline and at the end between the two groups. The composition of rate at baseline was calculated by Chi-square tests. The change of parameters equaled to the differences of the end values from the baseline values. The effects of fish oil supplement on anthropometric parameters, serum lipids, glucose, insulin, liver enzymes, cytokines in the participants in contrast to corn oil supplement were analyzed using ANCOVA with the change as the dependent variables, and adjusted with the correlating values at baseline (Model 1), or adjusted with the correlating values at baseline, age, gender and BMI (Model 2). Two-sided p values < 0.05 were considered as significant.
