# Supplementary material for: Fish Oil Supplements Lower Serum Lipids and Glucose in Correlation with a Reduction in Plasma Fibroblast Growth Factor 21 and Prostaglandin E2 in Nonalcoholic Fatty Liver Disease Associated with Hyperlipidemia: A Randomized Clinical Trial
Source: PLoS One. 2015 Jul 30;10(7):e0133496. doi: 10.1371/journal.pone.0133496 (PMC4520650; doi:10.1371/journal.pone.0133496)
Supplement: S1 Table — (DOCX) [file pone.0133496.s003.docx]

**Supporting Information Table S1 Effects of fish oil or corn oil on plasma free fatty acids spectra in study participants^a^**

|  | **Corn oil (n=34)** | | | **Fish oil (n=36)** | | | ***P*** | | |
| --- | --- | --- | --- | --- | --- | --- | --- | --- | --- |
|  | **Baseline** | **End** | **Change^b^** | **Baseline** | **End** | **Change** | **Baseline** | **ANCOVA^c^** |  |
| C14:0 (%) | 1.42±0.20 | 1.48±0.16 | 0.06±0.28 | 1.44±0.18 | 1.40±0.21 | -0.05±0.30 | 0.65 | 0.06 |  |
| C16:0 (%) | 25.90±2.96 | 25.95±3.09 | 0.05±4.21 | 26.07±2.85 | 26.30±3.00 | 0.23±4.33 | 0.81 | 0.63 |  |
| C18:0 (%) | 15.47±0.52 | 15.38±0.35 | -0.09±0.62 | 15.34±0.35 | 15.46±0.50 | 0.12±0.56 | 0.22 | 0.41 |  |
| C16:1 (%) | 2.42±0.23 | 2.42±0.24 | 0±0.33 | 2.40±0.23 | 2.37±0.23 | -0.02±0.34 | 0.72 | 0.42 |  |
| C18:1 (%) | 13.43±0.44 | 13.52±0.49 | 0.08±0.66 | 13.38±0.44 | 13.40±0.52 | 0.02±0.40 | 0.63 | 0.41 |  |
| C20:1 (%) | 0.61±0.07 | 0.61±0.06 | 0±0.08 | 0.61±0.07 | 0.61±0.08 | 0.01±0.10 | 0.79 | 0.96 |  |
| C18:2 (%) | 25.12±3.65 | 26.79±2.95 | 1.68±1.57 | 25.36±4.46 | 25.33±4.28 | -0.03±4.37 | 0.80 | 0.022 |  |
| C18:3 (%) | 2.43±0.25 | 2.42±0.23 | -0.01±0.35 | 2.42±0.22 | 2.43±0.24 | 0.01±0.35 | 0.90 | 0.85 |  |
| C20:3 (%) | 1.06±0.03 | 1.06±0.03 | 0±0.04 | 1.06±0.03 | 1.07±0.02 | 0.01±0.03 | 0.50 | 0.29 |  |
| C20:4 (%) | 4.25±0.90 | 4.26±0.82 | 0.01±1.14 | 4.17±0.81 | 4.32±0.93 | 0.16±1.21 | 0.68 | 0.74 |  |

^a^ Data are mean±SD. ^b^ Change is equal to the end values – the baseline values. ^c^ Adjusted by the baseline levels.
